# Exploring primary care health professionals’ perceived influence of their communication on HPV vaccine acceptance: Results from a national survey

**DOI:** 10.1371/journal.pone.0350507

**Published:** 2026-06-02

**Authors:** Olufeyisayo O. Odebunmi, Anna Ilyasova, Assanatou Bamogo, Yeshaben Patel, Colleen Higgins, Erin Laurie, Lisa N. Mansfield, Sachiko Ozawa, Stephanie B. Wheeler, Lisa P. Spees

**Affiliations:** 1 Department of Health Policy and Management, Gillings School of Global Public Health, University of North Carolina at Chapel Hill, Chapel Hill, North Carolina, United States of America; 2 Lineberger Comprehensive Cancer Center (LCCC), University of North Carolina at Chapel Hill, Chapel Hill, North Carolina, United States of America; 3 Department of Medicine, University of North Carolina at Chapel Hill, Chapel Hill, North Carolina, United States of America; 4 Department of Maternal and Child Health, Gillings School of Global Public Health, University of North Carolina at Chapel Hill, Chapel Hill, North Carolina, United States of America; 5 Division of Pharmaceutical Outcomes and Policy, Eshelman School of Pharmacy, University of North Carolina at Chapel Hill, Chapel Hill, North Carolina, United States of America; 6 Division of Practice Advancement and Clinical Education, Eshelman School of Pharmacy, University of North Carolina at Chapel Hill, Chapel Hill, North Carolina, United States of America; 7 School of Nursing, University of North Carolina at Chapel Hill, Chapel Hill, North Carolina, United States of America; Federal University Otuoke, NIGERIA

## Abstract

**Objective:**

To evaluate primary care health professionals’ (PCHPs) perspectives on how their communication influences HPV vaccine acceptance, based on the self-perception theory. And to examine the factors associated with PCHPs’ perceived influence on HPV vaccine acceptance.

**Methods:**

An online national survey was administered between May and July 2022 to PCHPs involved in HPV vaccination for children ages 9–12 years old. Survey items comprised of PCHPs’ demographics, clinic settings, and selected HPV communication measures, such as influence of vaccine communication, strategies and challenges, and past HPV vaccine communication training. Logistic regression models assessed factors associated with PCHPs’ perceived influence of their communication on HPV vaccine acceptance.

**Results:**

The majority of PCHPs felt their communication greatly influenced HPV vaccine acceptance. Compared to PCHPs who thought that communication had little or some influence on HPV vaccine acceptance, PCHPs who thought their communication greatly influenced HPV vaccine acceptance were more likely to: 1) use presumptive recommendations (aOR: 1.32; 95% CI: 1.11, 1.57), 2) report parental concerns about HPV vaccination promoting sexual activity (aOR:1.26, 95% CI: 1.06, 1.51), and 3) have had HPV vaccine communication training on how to address parental HPV vaccine hesitancy (aOR:1.25; 95% CI: 1.02, 1.54).

**Conclusion:**

This study demonstrates the pivotal role of vaccine communication in influencing HPV vaccine acceptance among PCHPs. Tailored vaccine communication training that involves the entire primary care team and equips them with effective communication techniques is essential to increase PCHP’s confidence in vaccine conversations and competence in making HPV vaccine recommendations. HPV vaccine communication should also include content that promotes the cancer preventive role of HPV vaccine and debunks the myth around HPV vaccination promoting sexual activity.

## Introduction

Human papillomavirus (HPV) vaccination is safe and effective in preventing HPV-related infections and cancers [1]. National guidelines routinely recommend HPV vaccine for adolescents ages 11–12 years, with vaccination starting as early as age 9 [1,2]. However, current HPV vaccination coverage is well below the Healthy People 2030 national target of 80%, with only 58.6% of adolescents ages 13–15 years receiving recommended doses [3]. Provider and healthcare system-level interventions are increasingly being used to increase HPV vaccination rates [4,5]. Examples of healthcare system-level strategies that have been used include HPV vaccine standing orders and reminder and recalls to parents with eligible children [6], while provider-level interventions include audit and feedback reports on HPV vaccination rates, provider assessment and feedback using the Centers for Disease Control and Prevention (CDC’s) Immunization Quality Improvement for Providers (IQIP), and provider-led HPV vaccine recommendations [4,5]. In particular, provider recommendation is the strongest predictor for HPV vaccine acceptance [7], especially when providers are trained in using evidence-based communication techniques like presumptive announcements [8,9]. A presumptive announcement is when a provider introduces HPV vaccine by making a statement that presumes the parent would want their child vaccinated (also known as a presumptive recommendation) [8,9]. A randomized controlled trial found that, compared to using a participatory style (i.e., dialogue between parent and provider about HPV vaccination), providers who used presumptive announcements had significantly higher HPV vaccine initiation among adolescents ages 11–12 years (5.4% difference) [8]. Further, other studies found that when additional tools—such as motivational interviewing, HPV vaccine fact sheets, or following up on HPV vaccine discussions after an initial refusal—are used with a presumptive recommendation, providers are more equipped to address parental HPV vaccine hesitancy, leading to increased HPV vaccine uptake [10–14].

Although US-based studies have demonstrated provider communication as a key determinant of HPV vaccine acceptance [7,15,16], few national-level studies have explored primary health care professionals’ (PCHPs), including providers’ (physicians, nurse practitioners or physician assistants) and clinical staff’s (registered nurses, licensed practical/vocational nurse, medical assistant or certified nursing assistant), perceptions on how their communication influences HPV vaccine uptake. Our study seeks to address this gap. Our study is based on self-perception theory where people infer their perceptions from their behaviors, especially when their feelings about a certain concept is unclear [17]. By observing PCHPs’ actions or experiences (such as past vaccination training, communication style, or communication challenges), we can infer their beliefs about HPV vaccine communication on vaccine acceptance. This knowledge could lead PCHPs to focus more on their future communications, and result in a larger impact on HPV vaccine acceptance. Hence, the purpose of this study is to: 1) evaluate PCHPs’ perceived influence of their communication on HPV vaccine acceptance, and 2) identify the factors associated with higher perceived influence. Understanding PCHPs’ perspectives about how their communication affects HPV vaccine acceptance will provide guidance on training needed to improve vaccine conversations with parents.

## Materials and methods

### Survey participants and procedures

An online national survey was developed by a multi-disciplinary team comprising of physicians, health services researchers, and behavioral health researchers. We administered the survey between May 2022 to July 2022 to PCHPs who were members of the Medscape Network directory, with about 60% of US physicians represented in the network [2]. Medscape curates a website database that provides health and medical care information, continuing education opportunities and research participation opportunities for healthcare professionals. Medscape invited members with the required training to indicate their interest in our survey, ensuring only active members would be contacted. Then, these potential eligible participants were contacted and recruited via email by Medscape and they completed an online eligibility screener. Medscape sent non-responders up to four email reminders to complete the survey. To ensure diversity across PCHP training types, our team used quotas in recruiting similar proportions of pediatricians, family medicine physicians, advanced practice providers (APPs), and clinical staff. Because of the rural-urban disparities in HPV vaccination, we oversampled PCHPs practicing in clinics located in rural counties categorized according to the USDA Rural-Urban Continuum Codes (RUCC) 4–9 [18]. Study approval was obtained by the Institutional Review Board at the University of North Carolina (#21-2829).

Eligible participants included 1) trained and licensed PCHPs in the US (pediatricians, family medicine physicians, advanced practice providers (APP) (nurse practitioners and physician assistants), and clinical staff (such as registered nurse (RN), licensed practical/vocational nurse (LPN, LVN), medical assistant (MA) or certified nursing assistant (CNA); and 2) PCHPs who had a role in HPV vaccination for children ages 9–12 years old. Roles in HPV vaccination included assessing HPV vaccination eligibility and status, announcing that a child is due for HPV vaccination, discussing the risks and benefits of HPV vaccine, addressing parents’ questions and concerns about HPV vaccination, or administering HPV vaccine to eligible children. We applied sampling quotas to ensure adequate representation and diversity across PCHPs (pediatricians, family medicine physicians, APPs, and clinical staff). The characteristics of our sample such as age, sex and race were compared with the Current Population Survey (CPS) and both results were comparable, justifying the representativeness of our survey respondents [19] (S1 Table). A total of 6,278 PCHPs indicated their interest in participating in our study. After excluding participants who did not meet our inclusion criteria (n = 1,179), did not respond to the survey invitation (n = 2,242), or did not complete the survey (n = 330), we had a final sample of 2,527 PCHPs who completed the online survey (response rate of 57%; American Association for Public Opinion Research Response Rate 3), after providing written informed consent. Participants were compensated with a $45 honorarium for their time, and their data were de-identified to all authors. More information on the survey instrument and administration process is published elsewhere [2].

### HPV vaccine communication measures

Cognitive interviews were conducted with 39 PCHPs to obtain their feedback and thoughts about the survey and to provide recommendations for modifying the survey. The responses from interview participants were compared to the intended survey goal and the survey items and instructions were revised to enhance understanding prior to its administration.

The survey included questions on HPV vaccine communication strategies used, challenges experienced in bringing up HPV vaccination, perceived influences on vaccine acceptance, and past HPV vaccination training received.

**Primary outcome.** The primary outcome, PCHPs’ level of communication influence is the extent to which PCHPs believed that their communication influenced parental HPV vaccine acceptance, which is based off the survey question- *“How much do you think the way you communicate with parents affect whether they accept HPV vaccination?”*. Response options ranged from “1-not at all” to “5-extremely.” “For our analyses, we initially categorized these response options into 3 groups: “0-little influence” (for “not at all” and “slightly”), “1-some influence” (for “moderately”) and “2-great influence” (for “very much” and “extremely”). However, due to the minimal differences between the first two groups, we combined them for the final analysis (S2 Table). Consequently, our main outcome was a binary variable with the categories, “0-little or some influence” and “1-great influence”.

**Key variables.** We assessed the following key variables: 1) PCHP medical training, 2) use of presumptive recommendations, 3) HPV vaccine communication challenges, and 4) past training on HPV vaccination. We categorized medical training as pediatricians, family medicine physicians, advanced practice providers (physician assistants, nurse practitioners, and clinical nurse specialists), and clinical staff (including registered nurses, licensed practical nurses, licensed vocational nurse, certified nurse assistants and medical assistants).

Our variable indicating use of presumptive recommendations was based on the survey item*- “Which HPV vaccine communication strategy do you most often use when bringing up HPV vaccine with parents of children ages 9-12?”*. Response options included: 1) I ask a question, such as: “How do you feel about HPV vaccine for your child today?”, 2) I assume routine vaccine acceptance and might say: “We’ll give vaccines today that protect against meningitis, HPV cancers, and whooping cough...”, 3) I bring up HPV vaccine only if asked about it. We created a binary variable called the use of presumptive recommendations for PCHPs coded as “0-No” (for PCHPs who selected response options 1 and 3) and “1-Yes” (for PCHPs who selected response option 2). Given the higher uptake of HPV vaccine among PCHPs who use presumptive recommendations [8,9], including this variable in our analyses could be informative in understanding PCHPs perceptions about how their communication influences parental vaccine acceptance.

For HPV vaccine communication challenges, we asked PCHPs the question*- “In the past year, what challenges have you encountered when you bring up HPV vaccine with parents of children ages 9-12? (Check all that apply)”* Response items included: 1) the belief that the child does not need HPV vaccine at the time, 2) parents’ concern about safety, 3) parents’ concern about promoting sexual activity, 4) long discussion time, 5) mistrust of providers or other clinic staff, and 6) mistrust of CDC vaccine recommendations. Six binary variables were created for each communication challenge. PCHPs experiences of challenges during HPV communication could provide some insights on how PCHPs view the impact of their communication on HPV vaccine acceptance.

For past HPV vaccine communication training, PCHPs were asked were asked, *“Have you received any training in HPV vaccination that included…”* Respondents could select from the following options: 1) continuing medical education (CME) credit, 2) how to bring up HPV vaccination, 3) how to address parent’s hesitancy, 4) roles of the full primary care team, 5) testimonial of HPV cancer survivors, 6) practice through role play, 7) webinar instruction, and 8) in-person instruction. Eight binary variables were created to represent each training item. PCHPs confidence in talking about HPV vaccine is improved through additional HPV vaccine education and communication training [20]. Hence including past HPV vaccination training in our analyses could help shed more light on PCHPs beliefs on the impact of their communication n vaccine acceptance.

**PCHP and clinic characteristics.** We obtained participants’ gender, race and ethnicity, and years of practice. Self-reported gender was categorized as man, woman, and other gender or prefer not to say. Self-reported race and ethnicity were grouped as White, Black, Asian, Hispanic, Multiracial (responses of two or more races), and other race or prefer not to say. Years of practice was operationalized as a categorical variable (0–9, 10–19 and 20 years and above). Characteristics of the main clinic in which PCHPs practiced included the number of patients ages 9–12 seen in a week, the number of providers at their clinic, and binary indicators for whether the clinic was part of a larger healthcare system, was a federally qualified health center, or was a public health department. We also determined the clinic’s rurality using RUCC [18]. Clinics in counties designated as RUCC 1–3 were categorized as non-rural while clinics in counties designated as RUCC 4–9 were categorized as rural [18]. We also included the clinic’s regional location, categorized as Northeast, South, Midwest, and West.

### Statistical analysis

We conducted descriptive statistics of the cohort, key variables (i.e., PCHP medical training, use of presumptive recommendations, HPV vaccine communication challenges, and past training on HPV vaccination), and the primary outcome, PCHPs’ level of communication influence. We ran bivariate and multivariable logistic regressions to evaluate how our key variables were associated with PCHPs’ level of communication influence on HPV vaccine acceptance, while controlling for PCHP (gender, race, ethnicity, years of practice) and clinic characteristics (clinic rurality, clinic region, clinic type and patient volume). All analyses were two-tailed with a critical alpha of 0.05 and were conducted using STATA 16.1 (College Station, TX).

## Results

### PCHPs’ demographics and clinic setting

The final analytic sample was 2,402 PCHPs using complete case analysis, [21] with data missingness due to non-responses to the survey item assessing PCHPs perceived HPV vaccine communication influence on HPV vaccine acceptance (missingness 5%). Most PCHPs were women (72%) and self-identified as White (66%). About a third of PCHPs had less than ten years of clinical practice, and 40% of PCHPs saw between 10–24 patients ages 9–12 in a week. Most of the clinics where PCHPs practiced were non-rural (91%) or part of a healthcare system (62%) (Table 1).

**Table 1:** Primary care health professional (PCHP) cohort characteristics.

	Total (N = 2,402)
	n (%)
**Level of communication influence on HPV vaccine acceptance**	
Little or some influence	1,089 (45)
Much influence	1,313 (55)
**Gender**	
Man	611 (25)
Woman	1,721 (72)
Another gender or prefer not to say^a^	70 (3)
**Race/ethnicity**	
White	1,587 (66)
Hispanic	93 (4)
Black	111 (5)
Asian	348 (14)
Multiracial^b^	88 (4)
Another race or prefer not to say	175 (7)
**Medical training**	
Pediatrician	648 (27)
Family med physician	537 (22)
Advanced practice providers^c^	586 (25)
Clinical staff^d^	631 (26)
**Number of patients seen in a week**	
<10	686 (29)
10-24	964 (40)
25+	752 (31)
**Years of practice**	
0-9	902 (38)
10-19	702 (29)
20+	798 (33)
**Healthcare system**	
No	904 (38)
Yes	1,498 (62)
**FQHC or health department**	
No	2,100 (87)
Yes	302 (13)
**Number of providers at clinic**	
<6	1,090 (46)
6-10	630 (26)
11+	682 (28)
**Clinic region**	
Northeast	480 (20)
Midwest	552 (23)
South	787 (33)
West	583 (24)
**Clinic rurality**	
Yes	218 (9)
No	2,184 (91)

Abbreviations: HPV- Human Papillomavirus; FQHC-Federally Qualified Health Center.

^a^Includes individual who identified as nonbinary or preferred not to report their gender.

^b^Includes individuals who identified with at least two racial/ethnic groups.

^c^Includes physician assistants, nurse practitioners, advanced practice nurses, and clinical nurse specialists.

^d^Includes registered nurses (RNs), licensed practical nurses, licensed vocational nurses, certified nursing assistants, and medical assistants.

### HPV vaccine communication measures

#### Vaccine influence.

Over half of PCHPs (55%, n = 1,313) felt that their communication had a great influence on HPV vaccine acceptance (Table 1). Slightly more than a third (37%, n = 885) felt that their communication had some influence, and only 8% (n = 204) felt that their communication had little influence (S2 Table).

#### Key variables.

**Fig 1:**
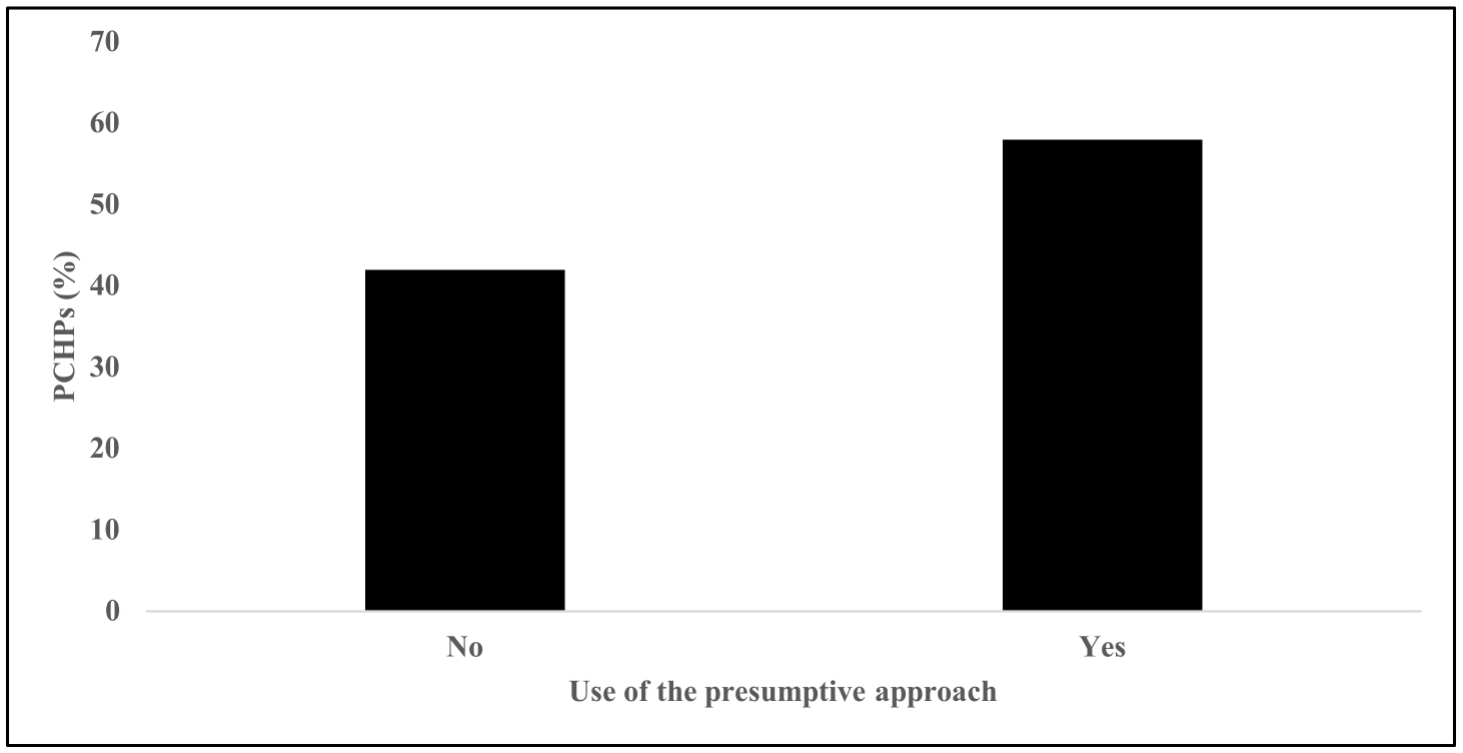
Use of presumptive recommendations among primary care health professionals (PCHPs).

**Fig 2:**
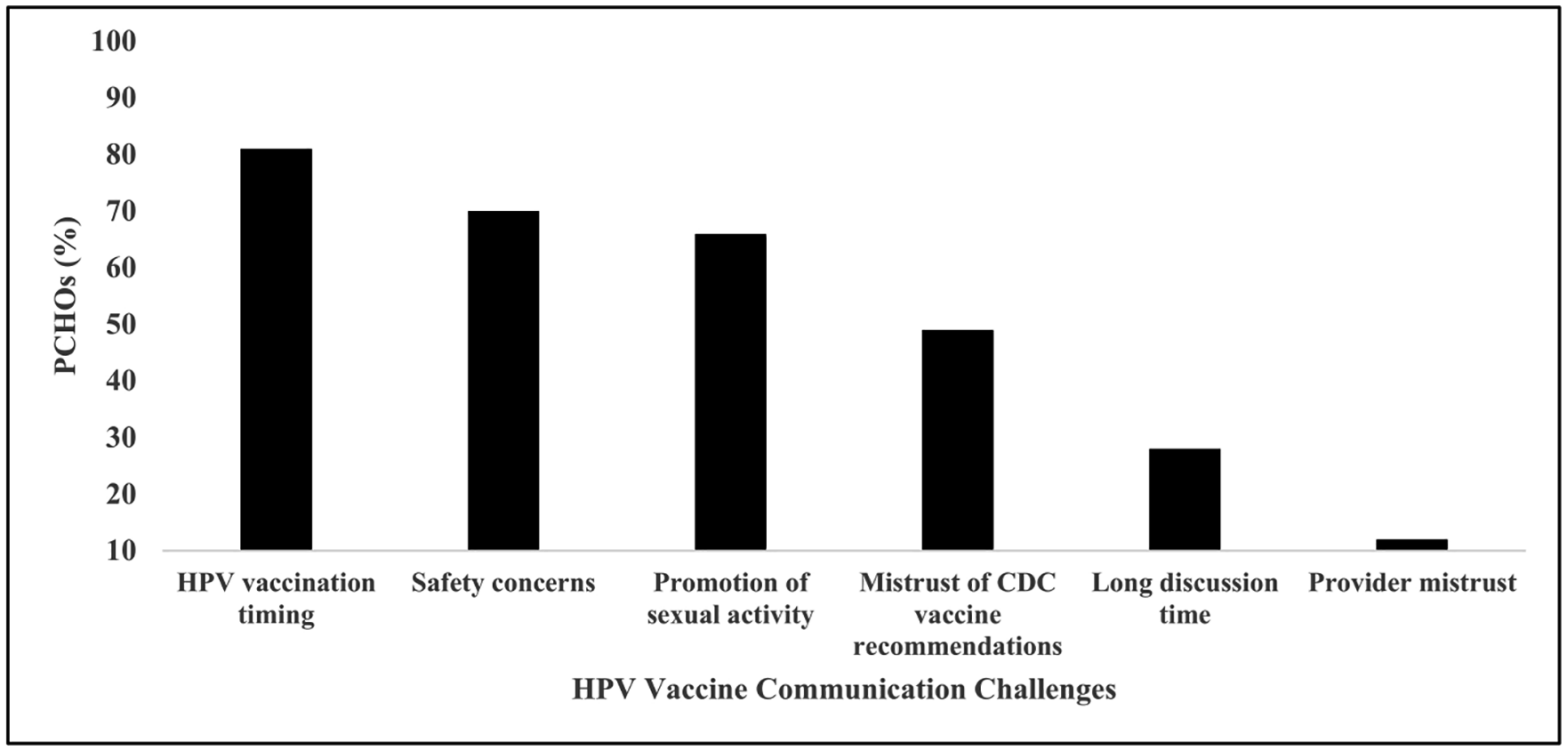
HPV vaccine communication challenges experienced by primary care health professionals (PCHPs).

There was approximately equal representation of PCHPs by medical training including pediatricians (27%), family medicine physicians (22%), advanced practice providers (24%), and clinical staff (26%). Almost two-thirds (58%) used presumptive recommendations when talking about HPV vaccine with parents (Fig 1). The most frequent challenge experienced by PCHPs was the parental belief that their child did not need HPV vaccination at the time of the clinic visit (81%; Fig 2), followed by safety concerns about HPV vaccination (70%), and promotion of sexual activity (66%). The least reported challenge was parental mistrust of providers (12%).

**Fig 3:**
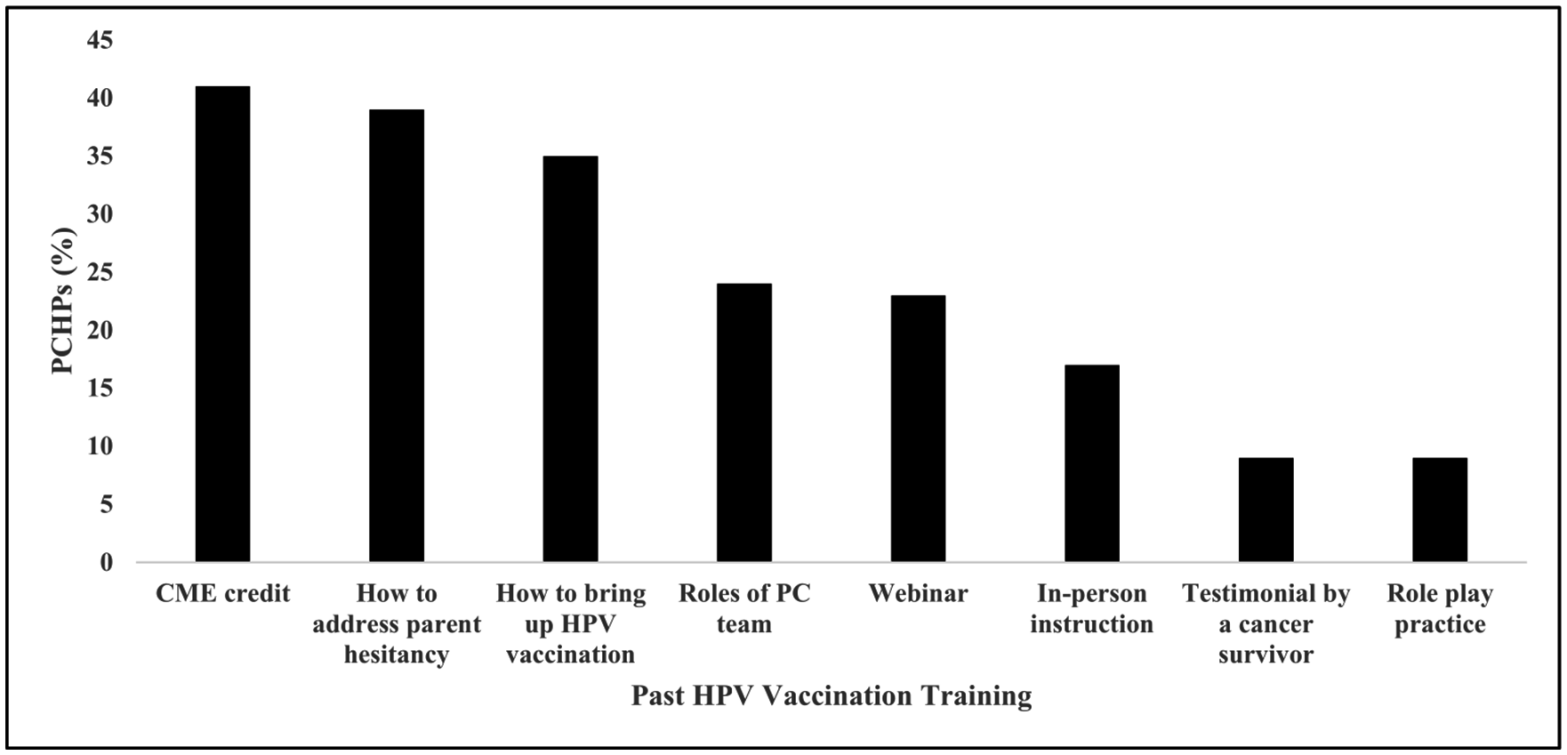
Past HPV vaccine training among primary care health professionals (PCHPs).

Approximately 40% of PCHPs had participated in HPV vaccination training where they received CME credits (41%; Fig 3) and on how to address parental HPV vaccine hesitancy (39%). About a quarter of PCHPs (24%) had participated in trainings that discussed the roles of the primary care team in HPV vaccination. Testimonials from a cancer survivor (9%) and practice through role play (9%) were the least common training features selected by PCHPs.

### Factors associated with PCHPs perceived communication influence on HPV vaccine acceptance

Our bivariate analyses showed that clinical staff were less likely to think their communication greatly influenced HPV vaccine acceptance compared to pediatricians (OR:0.75; 95% C.I: 0.60, 0.93). PCHPs who used presumptive recommendations or reported experiencing the challenge that HPV vaccine would promote sexual activity were more likely to believe their communication greatly influenced HPV vaccine acceptance. All indicators of past HPV vaccination training were associated with PCHPs being more likely to think their communication had a great influence on HPV vaccine acceptance (S3 Table).

**Fig 4:**
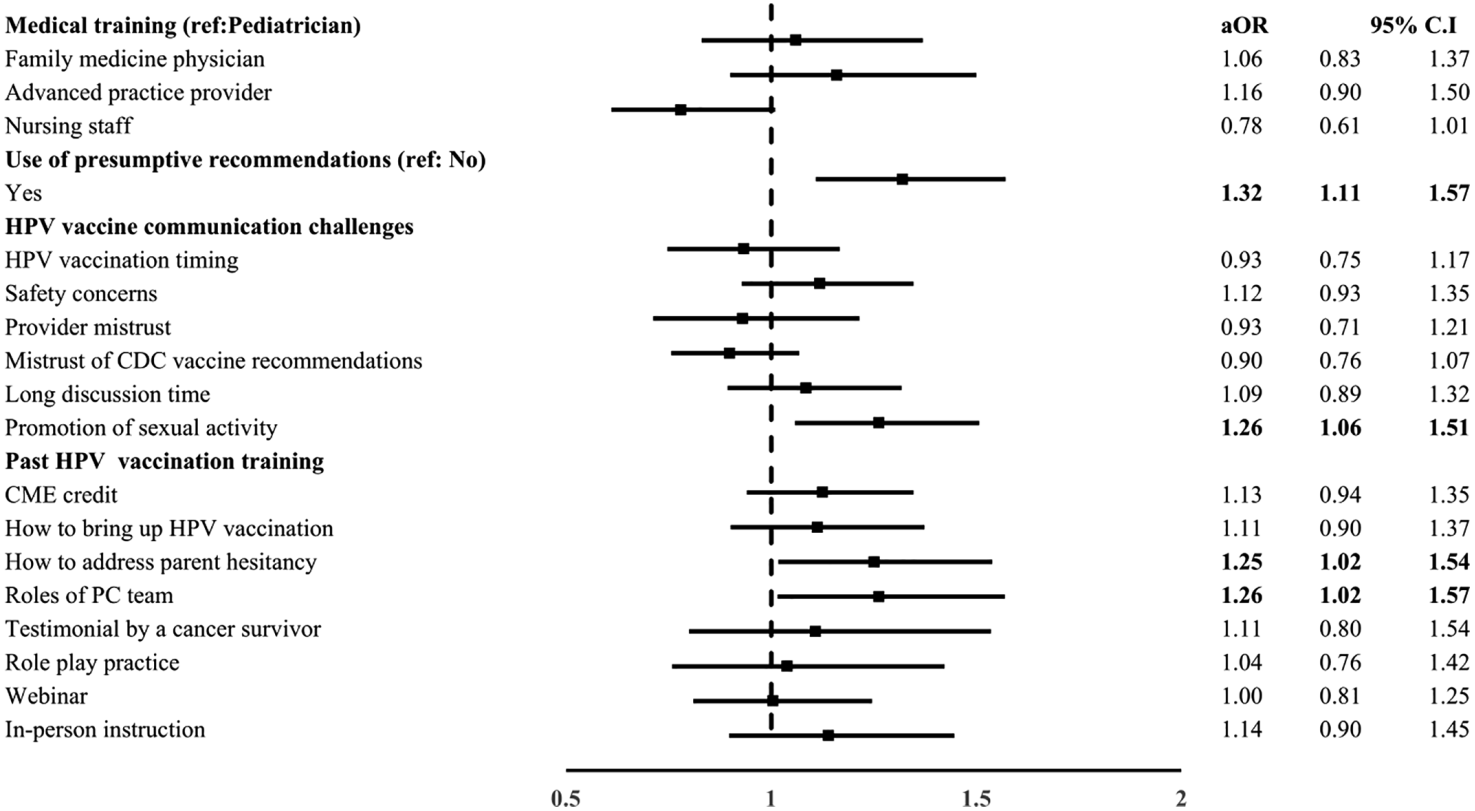
Associations with PCHPs’ belief that HPV vaccine communication greatly influences HPV vaccine acceptance. aOR-Adjusted odds ratio; CI-confidence interval; HPV-human papillomavirus; CME-continuing medical education; PC-primary care. CDC: Centers for disease control and prevention. Bolded values indicate p < 0.05. Model is adjusted for PCHPs’ race, gender, years of practice, practice region, medical training, clinic type and patient volume. The reference category for medical training was pediatricians and the reference categories for the use of presumptive recommendations, HPV vaccine communication challenges and past HPV vaccination training include those who responded “no”.

In multivariable analyses, medical training (family medicine physician, advanced practice provider, and nursing staff) and indicators of prior HPV vaccination training were no longer statistically significant*.* However, use of presumptive recommendations continued to be positively associated with PCHPs’ belief that their communication greatly influenced HPV vaccine acceptance (aOR: 1.32; 95% C.I: 1.11, 1.57). Additionally, PCHPs who reported encountering the HPV vaccine would promote sexual activity as a challenge with parents were more likely to think their communication had a great influence on HPV vaccine acceptance (aOR:1.26, 95% C.I: 1.06, 1.51). Finally, past HPV training indicators that continued to be associated with our primary outcome included training on 1) how to address parental hesitancy (aOR:1.25; 95% C.I: 1.02, 1.54), and 2) the roles of the primary care team in HPV vaccination (aOR:1.26, 95% C.I: 1.02, 1.57). The estimates for clinical staff were marginally significant (Fig 4).

## Discussion

From a national survey with PCHPs, we found that most PCHPs believed that their communication about HPV vaccination greatly influenced vaccine acceptance. PCHPs who used presumptive recommendations felt that their communication greatly influenced HPV vaccine acceptance compared to those who did not use presumptive recommendations. Similarly, PCHPs who had received HPV vaccination training on how to address parental hesitancy and the roles of a primary care team in HPV vaccination were also more likely to feel that their communication had a great influence on HPV vaccine acceptance.

HPV vaccine recommendations predict HPV vaccine acceptance [7,22]. In particular, the presumptive recommendation, where a provider introduces HPV vaccine by making a statement that assumes a parent would want to vaccinate their child for HPV [8,9], is an evidence-based communication style shown to increase HPV vaccination rates [8,9]. Thus, in our sample, PCHPs’ perception that their use of presumptive recommendation greatly influenced vaccine acceptance may reflect its demonstrated effectiveness in clinical practice. Also, our results demonstrate that most PCHPs engage with current evidence-based HPV vaccine communication techniques (i.e., presumptive recommendation). Past work reported that PCHPs felt that using a presumptive recommendation is time-saving and efficient [9, 23, 24]. Therefore, using presumptive language could provide several benefits to PCHPs, as this technique might reduce their discomfort in bringing up and administering the vaccine [9,20].

Previous HPV vaccine research has cited parental vaccine concerns including HPV vaccination promoting adolescent sexual activity [25,26]. Interestingly, our results show that PCHPs who faced parental hesitancy due to concerns about promoting sexual behavior were, in fact, also more likely to feel that their communication greatly influenced HPV vaccine acceptance. These PCHPs may feel more comfortable in addressing sensitive topics with parents. And because parental concern about sexual activity is a frequently cited reason for HPV vaccine refusal [25,26], PCHPs may anticipate this challenge and be better equipped to address it during HPV vaccine conversations. A systematic review found that providers who regularly had discussions about sexual activity with parents also reported experiencing minimal discomfort when these concerns arose during HPV vaccine conversations [27]. Hence, PCHPs should consider using communication strategies such as structured communication techniques (like presumptive recommendations) that enhance their comfortability in discussing sexual activity [28] (especially in debunking the belief that HPV vaccination could promote sexual activity) [29], building provider-parent rapport, and focusing on HPV vaccination as critical to cancer prevention [16,30].

Participating in HPV vaccination trainings is not only positively associated with PCHPs’ beliefs that their communication greatly influenced HPV vaccination, but these trainings are associated with increased PCHPs’ confidence and intentions to bring up HPV vaccination with parents and ultimately, increased HPV initiation rates [20,31]. In a cluster randomized trial involving a HPV vaccine communication training intervention, physicians, nurses, and physician assistants reported increased confidence in bringing up and discussing HPV vaccination with hesitant parents [32]. We demonstrate the importance of trainings on how to address parental hesitancy, an issue directly targeted by the three-step Announcement Approach [14]. While the first step of the Announcement Approach employs a presumptive recommendation, also known as a presumptive announcement, (which we found to be associated with PCHPs’ beliefs in their influence on HPV vaccine acceptance), the second step uses “connect and counsel”. Specifically, the “connect and counsel” step includes providing PCHPs with suggestions for how to effectively and efficiently respond to the most frequent reasons for parental hesitancy, including HPV vaccine misinformation [12,14]. Although previous research shows that Announcement Approach trainings among physicians increase HPV initiation [8], our results further exemplify AA trainings could benefit PCHPs from any level of medical training. Engaging all PCHPs involved in HPV vaccination in Announcement Approach training may increase their self-efficacy during HPV vaccine discussions, reinforce consistent vaccine messaging throughout clinic visits and enable parents to initiate vaccine conversations earlier in the visit prior to seeing the physician. Additionally, although results were not statistically significant for other HPV trainings, such as those involving CME credits, role play, and how to bring up HPV vaccination, these trainings remain important and should be included in PCHPs’ communication training. They ensure competent clinical practice through sustained learning, skill development, and professional growth. These trainings also prepare and equip PCHPs with the tools needed to effectively communicate with parents about HPV vaccine and complement approaches, such as the Announcement Approach [14]. In particular, the Announcement Approach training offers an opportunity for physicians and clinical staff to role play real conversations they can have with patients about HPV vaccine in a team-based training format that reflects their encounters in practice and can better prepare them to have conversations with parents.

Although the association with medical training was no longer statistically significant in our multivariable models, our findings may suggest that nursing staff feel their communication has limited influence on HPV vaccine acceptance*.* In previous literature, nurses and medical assistants have reported feeling less confident discussing HPV vaccination due to not knowing the correct vaccine dosing schedule [33]. This might be due to the differences in training and scope of practice between physician and non-physician trainees [34]. Future research needs to continue to explore expanding scope of practice in HPV vaccination communication between nurses and medical assistants, particularly since they may play an essential role in HPV vaccine communication

Our study has important limitations. First, due to the cross-sectional study design, we can only identify associations between variables and not causality. Future research should evaluate temporality regarding providers’ and clinical staff’s perspectives on their vaccine communication s, HPV vaccination training and use of the presumptive recommendations. Second, as with most survey-based studies, selection bias may be present, as the Medscape panel may only represent PCHPs who are more willing to participate in research or more engaged in continuing education activities. Additionally, response bias might be present in our sample, as participants may have reported perceptions viewed as generally acceptable, potentially introducing social desirability bias and resulting in biased estimates. Third, we were unable to determine how providers’ and clinical staff’s scope of practice for vaccinations impacted their perceptions of their communication influence on vaccine acceptance, their use of presumptive recommendations, or their ability to address communication challenges related to the HPV vaccine*.* Also, we do not have data on HPV vaccine uptake to validate our investigation of PCHPs’ perceived communication influence. Fourth, PCHPs’ perceptions are based on their interactions with parents willing to engage with healthcare providers. Consequently, our findings may not generalize to patients and families that do not interact with the healthcare system. However, even parents willing to engage with healthcare may be hesitant to vaccinate their children for HPV, making it critical for PCHPs to effectively communicate with them on the benefits of vaccinating their children. Fifth, we have no information on whether PCHPs in our sample practiced in locations affiliated with academic medical centers, as such locations might see patient populations that demographically differ from patients seen by PCHPs who practice in independent or community centers. Sixth, we used complete case analysis due to minimal missingness (5%) in our data, but there is the likelihood of reduced statistical power in our sample or decreased precision in our estimates. Lastly, our sample predominantly represented individuals working in urban settings and in large health care systems, meaning that our results may not be representative of rural or smaller clinics or clinics, thereby potentially limiting the generalizability of our results. Nevertheless, our national sample included PCHPs across all US regions, and we obtained perspectives from a diverse sample of PCHPs, including advanced practice providers, and nursing staff (medical assistants and certified nursing assistants), who play a critical role in HPV vaccine communication and acceptance.

## Conclusion

This study demonstrates the crucial role that communication plays in influencing HPV vaccine acceptance. Our findings highlight the value of presumptive recommendations and communication that emphasizes the cancer prevention benefits of HPV vaccine, addresses concerns about sexual activity, and maintains rapport with parents during HPV vaccine discussions. Our results also illustrate the need for system-level interventions to improve PCHPs’ communication, offering CME credits to engage PCHP in vaccine communication training and promoting use of the Announcement Approach in clinical practice. Finally, tailored training that involves the entire primary care team involved in HPV vaccine communication with parents (e.g., physicians, nurse practitioners, registered nurses, physician assistants, medical assistants and other clinical staff) is essential to increase PCHP’s confidence in vaccine conversations and improve the quality of HPV vaccine communication with parents in primary care. Future research should employ longitudinal studies to investigate the relationship between PCHPs’ perceived influence of their HPV vaccine communication on actual parental HPV vaccine acceptance. In addition, qualitative studies are needed to further explore the communication experiences of these PCHPs. Future work should evaluate temporality in providers’ and clinical staff’s perspectives on vaccine communication, HPV vaccination training and use of presumptive recommendations.

## Supporting information

S1 Table Sample characteristics compared with the Current Population Survey (CPS) on white race, female sex, and average age. (DOCX)

S2 Table Primary care health professional (PCHP) cohort characteristics, overall and stratified based on perceived influence of PCHP communication on HPV vaccine acceptance. (DOCX)

S3 Table Bivariate associations between primary care health professionals (PCHPs) and the perceived influence of PCHPs’ communication on HPV vaccine acceptance. (DOCX)
