# Supplementary material for: Exploring primary care health professionals’ perceived influence of their communication on HPV vaccine acceptance: Results from a national survey
Source: PLoS One. 2026 Jun 2;21(6):e0350507. doi: 10.1371/journal.pone.0350507 (PMC13229356; doi:10.1371/journal.pone.0350507)
Supplement: S1 Table — (DOCX) [file pone.0350507.s001.docx]

| **S1 Table: Sample characteristics compared with the Current Population Survey (CPS) on white race, female sex, and average age** | | | | | | |
| --- | --- | --- | --- | --- | --- | --- |
|  | **White** | | **Female** | | **Average age** | |
|  | **P01** | **CPS** | **P01** | **CPS** | **P01** | **CPS** |
| Physicians | 62% | 69% | 55% | 43% | 47 | 53 |
| Advanced practitioners^1^ | 76% | 85% | 84% | 92% | 43 | 43 |
| Registered nurses | 78% | 68% | 89% | 98% | 46 | 48 |
| Nursing staff^2^ | 70% | 53% | 91% | 89% | 43 | 40 |
| *Notes:* ^1^ Includes physician assistants and nurse practitioners; ^2^ includes certified nursing assistants, medical assistants, licensed vocational nurses, and licensed practical nurses | | | | | | |
