# Supplementary material for: Exploring primary care health professionals’ perceived influence of their communication on HPV vaccine acceptance: Results from a national survey
Source: PLoS One. 2026 Jun 2;21(6):e0350507. doi: 10.1371/journal.pone.0350507 (PMC13229356; doi:10.1371/journal.pone.0350507)
Supplement: S2 Table — (DOCX) [file pone.0350507.s002.docx]

**S2 Table.** **Primary care health professional (PCHP) cohort characteristics, overall and stratified based on perceived influence of PCHP communication on HPV vaccine acceptance**

|  | **Total** | **little influence** | **some influence** | **great influence** |
| --- | --- | --- | --- | --- |
|  | N=2,402  n (%) | (n=204)  n (%) | (n=885)  n (%) | (n=1,313)  n (%) |
| **Gender** |  |  |  |  |
| Man | 611 (25) | 45 (22) | 230 (26) | 336 (26) |
| Woman | 1,721 (72) | 152 (75) | 629 (71) | 940 (72) |
| Another gender | 70 (3) | 7 (3) | 26 (3) | 37 (3) |
| **Race/ethnicity** |  |  |  |  |
| White | 1,587 (66) | 141 (69) | 591 (67) | 855 (65) |
| Hispanic | 93 (4) | 11 (5) | 32 (4) | 50 (4) |
| Black | 111 (5) | 12 (6) | 26 (3) | 73 (6) |
| Asian | 348 (14) | 21 (10) | 136 (15) | 191 (15) |
| Another race | 175 (7) | 15 (7) | 64 (7) | 96 (7) |
| Multiracial | 88 (4) | 4 (2) | 36 (4) | 48 (4) |
| **Training specialty** |  |  |  |  |
| Pediatrician | 648 (27) | 47 (23) | 226 (26) | 375 (29) |
| Family med physician | 537 (22) | 36 (18) | 213 (24) | 288 (22) |
| APP | 586 (24) | 47 (23) | 209 (24) | 330 (25) |
| Clinical staff | 631 (26) | 74 (36) | 237 (27) | 320 (24) |
| **Number of patients seen in a week** |  |  |  |  |
| <10 | 686 (29) | 55 (27) | 282 (32) | 349 (27) |
| 10-24 | 964 (40) | 89 (44) | 358 (40) | 517 (39) |
| 25+ | 752 (31) | 60 (29) | 245 (28) | 447 (34) |
| **Years of practice** |  |  |  |  |
| 0-9 | 902 (38) | 93 (46) | 353 (40) | 456 (35) |
| 10-19 | 702 (29) | 50 (25) | 265 (30) | 387 (29) |
| 20+ | 798 (33) | 61 (30) | 267 (30) | 470 (36) |
| **Healthcare system** |  |  |  |  |
| No | 904 (38) | 71 (35) | 323 (37) | 510 (39) |
| Yes | 1,498 (62) | 133 (65) | 562 (64) | 803 (61) |
| **FQHC or health department** |  |  |  |  |
| No | 2,100 (87) | 180 (88) | 777 (88) | 1,143 (87) |
| Yes | 302 (13) | 24 (12) | 108 (12) | 170 (13) |
| **Use of presumptive recommendations** |  |  |  |  |
| No | 924 (38) | 90 (44) | 376 (42) | 458 (35) |
| Yes | 1,478 (62) | 114 (56) | 509 (58) | 855 (65) |
| **Number of providers at clinic** |  |  |  |  |
| <6 | 1,090 (45) | 102 (50) | 419 (47) | 569 (43) |
| 6-10 | 630 (26) | 44 (22) | 228 (26) | 358 (27) |
| 11+ | 682 (28) | 58 (28) | 238 (27) | 386 (29) |
| **Clinic region** |  |  |  |  |
| Northeast | 480 (20) | 34 (17) | 170 (19) | 276 (21) |
| Midwest | 552 (23) | 48 (24) | 215 (24) | 289 (22) |
| South | 787 (33) | 78 (38) | 270 (31) | 439 (33) |
| West | 583 (24) | 44 (22) | 230 (26) | 309 (24) |
| **Clinic rurality** |  |  |  |  |
| No | 2,184 (91) | 177 (87) | 806 (91) | 1,201 (91) |
| Yes | 218 (9) | 27 (13) | 79 (9) | 112 (9) |
| **HPV vaccine communication challenges** |  |  |  |  |
| HPV vaccination timing | 1,986 (83) | 165 (81) | 731 (83) | 1,090 (83) |
| Safety concerns | 1,689 (70) | 142 (70) | 611 (69) | 936 (71) |
| Promotion of sexual activity | 1,603 (67) | 126 (62) | 563 (64) | 914 (70) |
| Mistrust of CDC vaccine recommendations | 1,184 (49) | 98 (48) | 450 (51) | 636 (48) |
| Long discussion time | 657 (27) | 37 (18) | 244 (28) | 376 (29) |
| Provider mistrust | 281 (12) | 24 (12) | 107 (12) | 150 (11) |
| **Past HPV vaccination training** |  |  |  |  |
| Continuing medical education (CME) credit | 995 (41) | 81 (40) | 326 (37) | 588 (45) |
| How to address parent hesitancy | 966 (40) | 74 (36) | 300 (34) | 592 (45) |
| How to bring up HPV vaccination | 880 (37) | 68 (33) | 277 (31) | 535 (41) |
| Roles of the primary care team | 586 (24) | 48 (24) | 175 (20) | 363 (28) |
| Webinar | 558 (23) | 48 (24) | 179 (20) | 331 (25) |
| In-person instruction | 421 (18) | 39 (19) | 127 (14) | 255 (19) |
| Practice through role play | 225 (9) | 13 (6) | 73 (8) | 139 (11) |
| Testimonial of a cancer survivor | 207 (9) | 21 (10) | 55 (6) | 131 (10) |
|  |  |  |  |  |
