# Supplementary material for: Exploring primary care health professionals’ perceived influence of their communication on HPV vaccine acceptance: Results from a national survey
Source: PLoS One. 2026 Jun 2;21(6):e0350507. doi: 10.1371/journal.pone.0350507 (PMC13229356; doi:10.1371/journal.pone.0350507)
Supplement: S3 Table — (DOCX) [file pone.0350507.s003.docx]

**S3 Table. Bivariate associations between primary care health professionals (PCHPs) and the perceived influence of PCHPs’ communication on HPV vaccine acceptance**

|  | **n/N** | **Odds Ratio** | **95% confidence interval** |
| --- | --- | --- | --- |
| **Medical Training (Ref: Pediatrician)** |  |  |  |
| Family medicine physician | 288/557 | 0.84 | 0.67, 1.06 |
| Advanced practice provider | 330/603 | 0.94 | 0.75, 1.18 |
| Clinical staff  **Use of presumptive recommendation (Ref: No)** | 320/701 | 0.75 | **0.60, 0.93** |
| Yes | 855/1478 | 1.4 | **1.18, 1.65** |
| **HPV communication challenges** |  |  |  |
| HPV vaccination timing | 1090/1986 | 1.05 | 0.85, 1.03 |
| Safety concerns | 936/1689 | 1.11 | 0.93, 1.32 |
| Provider mistrust | 150/281 | 0.94 | 0.73, 1.21 |
| Mistrust of CDC vaccine recommendations | 636/1184 | 0.93 | 0.79, 1.09 |
| Long discussion time | 376/657 | 1.15 | 0.96, 1.38 |
| Promotion of sexual activity | 914/1603 | 1.33 | **1.12, 1.58** |
| **Past HPV training** |  |  |  |
| Continuing medical education (CME) credit | 588/995 | 1.36 | **1.15, 1.60** |
| How to bring up HPV vaccination | 535/880 | 1.48 | **1.25, 1.76** |
| How to address parent hesitancy | 592/966 | 1.57 | **1.33, 1.85** |
| Roles of the primary care team | 363/586 | 1.48 | **1.23, 1.80** |
| Testimonial by a cancer survivor | 131/207 | 1.48 | **1.10, 1.98** |
| Role play practice | 139/225 | 1.38 | **1.04, 1.83** |
| Webinar | 331/558 | 1.28 | **1.06, 1.55** |
| In-person instruction | 255/421 | 1.34 | **1.08, 1.66** |

“N” refers to the total number of PCHPs in the row; “n” refers to those who perceived their communication had a great influence on vaccine acceptance
